# Main Chemical Components, Activity and Mechanism of Repellence of *Cyperus esculentus* Essential Oil Against *Tribolium confusum*

**DOI:** 10.3390/molecules30030631

**Published:** 2025-01-31

**Authors:** Xu Feng, Cheng-Bin Shan, Jian-Nan Ma, Yue Ma, Na Li, De-Jian Zhang, Zhan-Yuan Lu, Chao-Mei Ma

**Affiliations:** 1 Key Laboratory of Herbage & Endemic Crop Biology of Ministry of Education, School of Life Sciences, Inner Mongolia University, Hohhot 010070, China; 21908003@mail.imu.edu.cn (X.F.); chengbin.shan@foxmail.com (C.-B.S.); 20160021@immu.edu.cn (J.-N.M.); my1020458810@163.com (Y.M.); lina15148040927@163.com (N.L.); zhangdejian00@163.com (D.-J.Z.); 2 Department of Traditional Chinese Medicine Resources and Development, College of Pharmacy, Inner Mongolia Medical University, Hohhot 010110, China; 3 Inner Mongolia Academy of Agricultural and Animal Husbandry Sciences, Hohhot 010031, China; lzhy2811@163.com

**Keywords:** *Tribolium confusum*, *Cyperus esculentus*, pest repellent, essential oil, cyperotundone, cyperene, pest enzymes

## Abstract

*Tribolium confusum* is a major stored-product pest that exhibits resistance to chemically synthesized pest repellents. This study investigated the potential of essential oil (EO) extracted from the roots of *Cyperus esculentus* as a natural alternative for pest management. The EO was obtained through steam distillation, and its chemical composition was elucidated using gas chromatography–mass spectrometry. The primary compounds, cyperotundone and cyperene, were further isolated from the EO through silica gel column chromatography. The efficacy of the EO and its isolated compounds as pest repellents was evaluated against a flaxseed pest, which was identified as *T. confusum* through DNA sequence analysis. The results demonstrated that at 86.12 μg/cm^2^, the EO and its two main components maintained significant repellent activity for up to 24 h. In contrast, the effectiveness of the positive control, N, N-diethyl-3-methylbenzamide (DEET) declined rapidly after 8 h. At 16 h, the repellent activity of the EO and one of its main components, cyperotundone, was significantly greater than that of DEET. Furthermore, at a lower concentration of 43.06 μg/cm^2^, cyperotundone’s repellent activity was significantly stronger than DEET’s at 16 h. Additionally, cyperotundone outperformed DEET significantly from 4 to 16 h at 21.53 μg/cm^2^ and at 16 h at 10.76 μg/cm^2^. Among the two compounds, cyperotundone exhibited a longer-lasting repellent effect compared to cyperene, which is consistent with the lower evaporation rate of cyperotundone. Biochemical assays revealed that exposure to the EO of *C. esculentus* and its major compounds significantly reduced (*p* < 0.05) the activities of acetylcholinesterase and glutathione-S-transferase in *T. confusum*. Molecular docking experiments indicated that the compounds could bind to olfactory receptors with low binding energies. qRT-PCR analysis revealed that the EO and its two compounds significantly altered (*p* < 0.05) the expression levels of odorant receptor genes in the pest. These findings suggest that the repellent action of *C. esculentus* EO and its major compounds on *T. confusum* may be mediated through the modulation of the pest’s olfactory system, as well as by inhibiting essential enzymatic activities in the pests. This research contributes valuable insights into the development of sustainable, long-lasting, and eco-friendly pest repellents, harnessing the potential of the rich botanical resource *C. esculentus*.

## 1. Introduction

Pests pose a significant challenge to global grain storage [1]. Species of the *Tribolium* genus (Coleoptera: Tenebrionidae) are particularly harmful to stored rice, flour, corn, peanuts, flaxseed kernels, and more. *T. confusum* Jacquelin du Val, a species found worldwide, is a primary cause of damage to stored products [2]. Its consumption, damage, and contamination, along with its ability to foster mold growth, can lead to product spoilage and failure to meet regulatory standards, resulting in substantial economic losses [3]. Chemical insect repellents and pesticides, such as phosphine, are commonly used to manage stored-product pests effectively [4]. However, the overuse of these chemicals has led to resistance in *T. confusum* [5], with additional negative impacts including environmental and health risks [6]. Plant-based pest repellents and pesticides offer the benefits of lower toxicity and environmental compatibility, making them valuable alternatives to chemically synthesized products in the prevention of *T. confusum* [7,8].

*Cyperus esculentus* var. sativus Boeckeler (Cyperales: Cyperaceae) is globally cultivated primarily for its nutritious tubers, commonly known as tiger nuts [9,10]. Notably, regions where *C. esculentus* grows are often free from pest infestations, hinting at the presence of natural pesticide or pest-repellent compounds within the plant. A variety of bioactive constituents have been isolated from both the tubers and leaves of *C. esculentus*, including stigmasterol, fatty acids, glycerol esters, 4-chlorobutyl oleate, oleamide, tyramine, N-feruloyltyramine, myricetin, quercetin, orientin, and other flavonoids, as well as caffeoylquinic acids [10,11]. These compounds have been documented to possess potent antioxidant properties [12,13] and hepatoprotective effects [11,14]. Despite this, the specific pest-repellent components of *C. esculentus* remain largely unexplored.

In our preliminary experiments on different parts of *C. esculentus*, the plant roots showed repellent effects, particularly at the 8 h exposure mark, where their effect was statistically stronger than that of the leaves and stems. Additionally, at the 16 h exposure mark, the roots exhibited a statistically stronger repellent effect in flour compared to the tubers (Figures S1 and S2 and Table S1). The distinct aroma emanating from the roots of *C. esculentus* suggests the presence of essential oil (EO). Plant EOs are blends of volatile substances, predominantly composed of monoterpenes and sesquiterpenes, some of which were known for their potential to act as pesticides or as repellents to pests [15,16]. The low toxicity and high volatility of these EOs could mitigate concerns regarding environmental contamination and chemical residue issues on stored grains [7,8].

The objective of this study was to assess the efficacy and mechanism of the EO and its constituent compounds derived from the roots of *C. esculentus* as repellents against *T. confusum*, thereby establishing a scientific foundation for the application of *C. esculentus* as a natural source of pest repellents.

## 2. Results

### 2.1. Components of the Essential Oil of C. esculentus Roots

**Figure 1:**
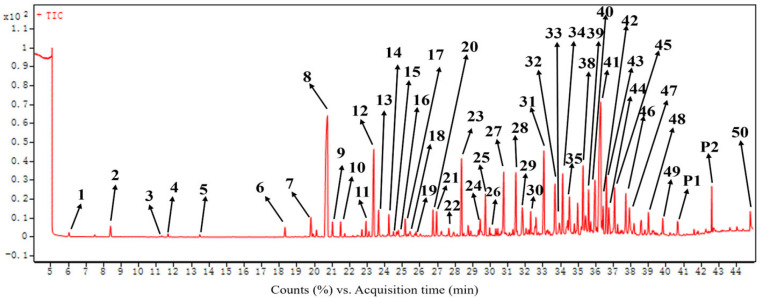
GC-MS chromatogram of *C. esculentus* root essential oil.

The EO extracted from the roots of *C. esculentus* was subjected to gas chromatography–mass spectrometry (GC-MS) analysis (Figure 1). The retention times of each constituent compound were compared against those of n-alkanes to calculate the retention index (RI). Preliminary identification of the volatile components was achieved by referencing the compound library within the GC-MS system and by correlating the RI values with literature-reported data, as detailed in Table 1. A total of 50 compounds were tentatively identified within the EO, making this the most comprehensive report on the volatile components of *C*. *esculentus* roots. Monoterpenoids and sesquiterpenoids constituted more than 90% of the total composition in the EO. The primary compounds of the EO were two sesquiterpenoids, cyperene (**8**) and cyperotundone (**41**), accounting for 13.54% and 13.50% of the total composition, respectively. These concentrations markedly exceeded those of other compounds detected, each of which was less than 5.5%.

### 2.2. Isolation and Structural Confirmation of the Two Main Compounds in the Essential Oil

**Figure 2:**
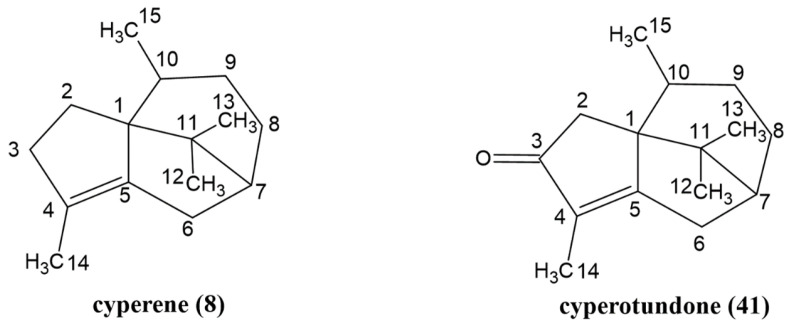
Structures of the main compounds, **8** and **41**, in the essential oil of *C. esculentus* roots.

The two main compounds of *C. esculentus* root EO were isolated using silica gel chromatography methods. Compound **8**, with an *m*/*z* of 204 on GC-MS, was obtained as a white powder, while compound **41,** with an *m*/*z* of 218 on GC-MS, was obtained as an oil. In the NMR spectra (Table S2 and Figures S3–S6), both compounds displayed signals of three singlets and one doublet, each with an integral of three protons, indicating the presence of four methyl groups in each compound’s structure. One of the singlet signals appeared at 1.64/1.68 ppm, which is characteristic of a methyl group attached to an olefinic carbon. The doublet signals at 0.83/0.58 ppm indicate that one of the methyl groups is attached to a tertiary carbon. By analyzing the ^1^H-^1^H COSY spectra (Figures S4 and S6) and comparing the ^1^H-NMR data with values reported in the literature [17,18], compounds **8** and **41** were identified as cyperene and cyperotundone, respectively. The structures of these two major sesquiterpenoids are depicted in Figure 2.

### 2.3. Pest-Repellent Activities of C. esculentus Root Essential Oil and the Main Compounds

The efficacy of pest repellency for samples with varying concentrations and exposure durations was quantified and is detailed in Table 2. At the highest concentration tested, which was 86.12 μg/cm^2^, all samples—namely the *C. esculentus* root EO, cyperene, cyperotundone, and the positive control N, N-diethyl-3-methylbenzamide (DEET)—showed more than 50% pest-repellent effects over the exposure period of 1 to 8 h. The EO and its two constituents maintained substantial repellent activity for up to 24 h. In contrast, the efficacy of DEET diminished rapidly after 8 h. At 16 h, the repellent activities of the EO and one of its main constituent compounds, cyperotundone, were statistically significant compared to DEET. At a lower concentration of 43.06 μg/cm^2^, both the EO and its compounds maintained more than 50% pest-repellent effects over the exposure period of 1 to 16 h, and the activity of cyperotundone was significantly stronger than that of DEET at 16 h. Moreover, the effects of cyperotundone were significantly stronger than those of DEET at 4–16 h at 21.53 μg/cm^2^ and at 16 h at 10.76 μg/cm^2^.

A three-way analysis of variance (ANOVA) was performed to investigate the effects of time, concentration, and group on the pest-repellent activity. The results presented in Table S3 and Figure S7 showed that the repellent activity of *C. esculentus* EO and its main compounds was superior to that of DEET in terms of the low concentration and temporal efficacy.

**Figure 3:**
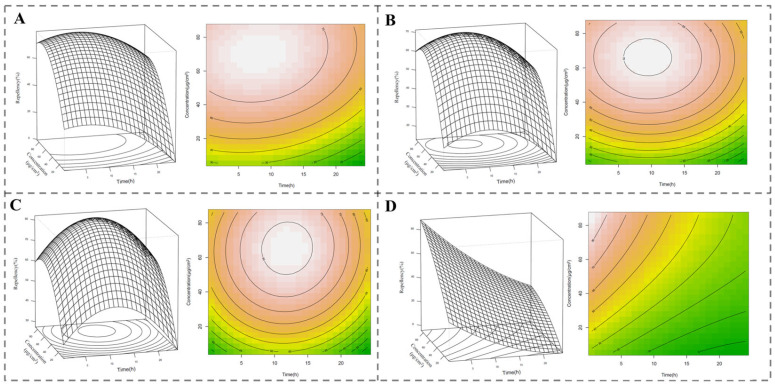
Simulated changes in spatial repellent activity with concentration and exposure interval using response surface methodology. (**A**) *C. esculentus* root essential oil; (**B**) cyperene; (**C**) cyperotundone; (**D**) DEET. The pest repellent data were analyzed with a response surface model using the rsm (version 2.10.5) package [19] in R and the results were visualized using the contour and persp functions.

Changes in spatial repellent activity, simulated with varying concentrations and exposure intervals using response surface methodology [19], are shown in Figure 3. The maximum repellent activities of the EO (79.85%), cyperene (71.27%), and cyperotundone (82.09%) appear at 8.09 h with 70.62 μg/cm^2^, 9.52 h with 65.73 μg/cm^2^, and 11.89 h with 64.91 μg/cm^2^, respectively, while the maximum repellent activity of the positive control, DEET (88.28%), appears at 0.5 h with 86.12 μg/cm^2^.

The results suggests that *C. esculentus* EO and the main compounds may offer more effective protection against pests over time and at varying concentrations compared to the synthetic repellent DEET.

### 2.4. Inhibitory Activity on the Pest Enzymes

**Figure 4:**
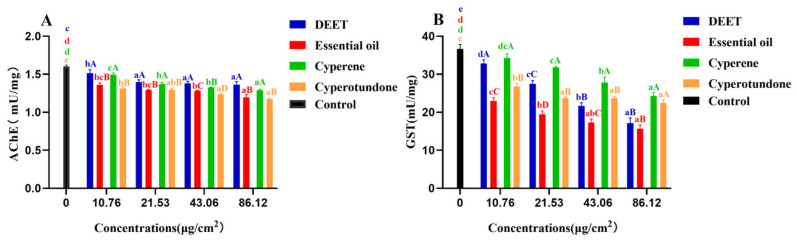
Inhibitory activities (mean ± standard deviations) of the essential oil, its main compounds and the positive control DEET on the pest enzymes AChE (**A**) and GST (**B**). Different letters indicated the existence of significantly difference by one-way ANOVA followed by Tukey’s HSD test (*p* < 0.05). a, b, c, d, comparison between different concentrations of the same sample; A, B, C, D, comparison between different samples of the same concentration.

After 24 h exposure to the sample, the activities of the pest enzymes, acetylcholinesterase (AChE) and glutathione S-transferase (GST), were quantified, and the results are shown in Figure 4 and Table S4. The EO extracted from the roots of *C. esculentus*, along with its two main compounds, cyperene and cyperotundone, significantly inhibited the activities of pest AChE and GST relative to those of the control group (*p* < 0.05). These findings indicate that the inhibition of AChE and GST activities is a component of the pest-repellent mechanism attributed to the EO from *C. esculentus* roots and its two primary compounds.

### 2.5. Docking Results of Main Compounds of C. esculentus Root EO with Odorant Receptor Proteins in the Pest

**Figure 5:**
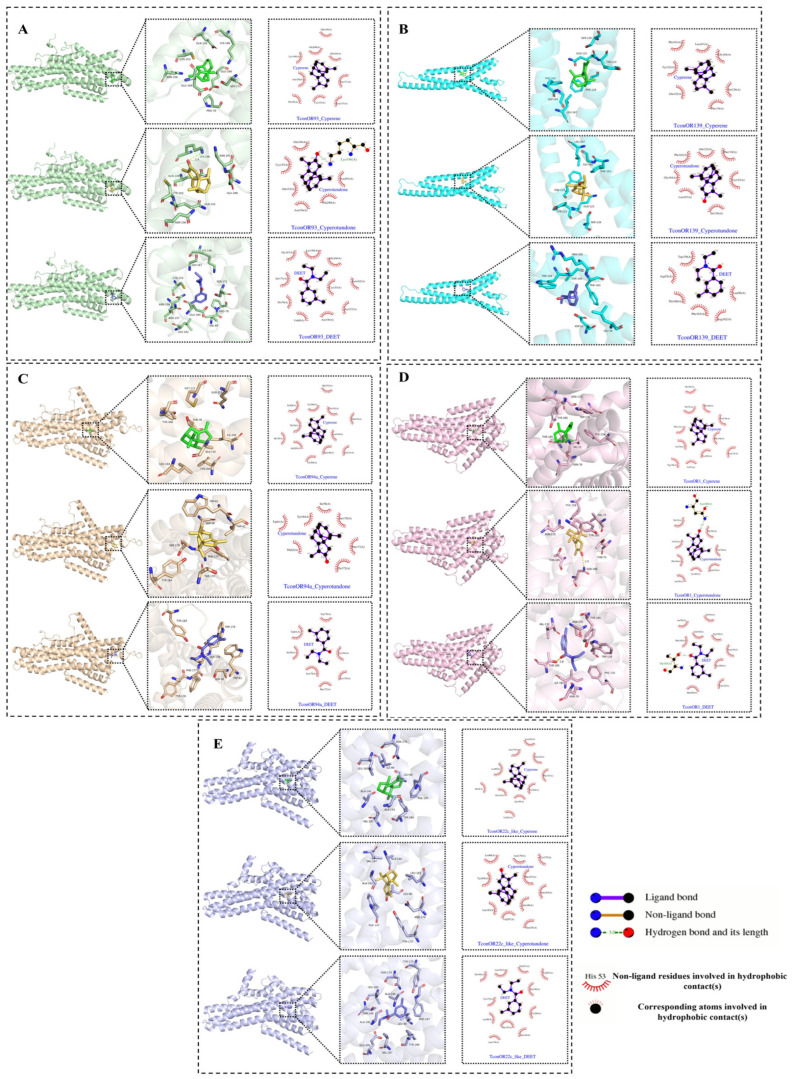
Visualization of the docking results of the EO main compounds and DEET with odorant receptor proteins in the pest. TconOR93 (**A**). TconOR139 (**B**). TconOR94a (**C**). TconOR1 (**D**). TconOR22c-like (**E**).

Some odorant receptors in *Tribolium confusum* have been identified as being involved in the response to the repellent activity of the volatile terpene compound limonene [5]. The interactions of these olfactory receptors (TconOR93, TconOR139, TconOR94a, TconOR1, and TconOR22c-like) with cyperene, cyperotundone, and DEET were thus investigated using molecular docking. The results are presented in Table 3; the binding affinities of cyperene and cyperotundone with the five target proteins were found to be higher than DEET. Cyperene and DEET demonstrated the lowest binding energies with the TconOR93 protein, at −7.9 kcal/mol and −7.0 kcal/mol, respectively. Cyperotundone showed the lowest binding energy with the TconOR139 protein, at −8.2 kcal/mol. Based on the binding modes visualized in Figure 5, the amino acid residues involved in the interactions between the small-molecule ligands and the protein pocket are clearly observed. Cyperene, cyperotundone, and DEET all form hydrophobic interactions with the amino acids in the protein pocket. Notably, cyperotundone also forms strong hydrogen bonds with the amino acid residues LYS-146 (2.87 Å) of TconOR93 and ASN-188 (2.92 Å) of TconOR1, while DEET forms a strong hydrogen bond with the amino acid residue THR (2.81 Å) of TconOR1. These interactions are crucial for anchoring the small molecules within the protein pocket, enhancing their stability at the binding site, and contributing to the formation of stable complexes.

The binding of a molecule to a receptor protein can lead to significant changes in gene expression by activating intracellular signaling pathways. These pathways ultimately influence the activity of transcription factors, which in turn control the transcription of specific genes. Therefore, the effects of these compounds on the expression of the corresponding odorant receptor genes in the pest need to be examined.

### 2.6. Effects on the Expression of Odorant Receptor Genes in the Pest

**Figure 6:**
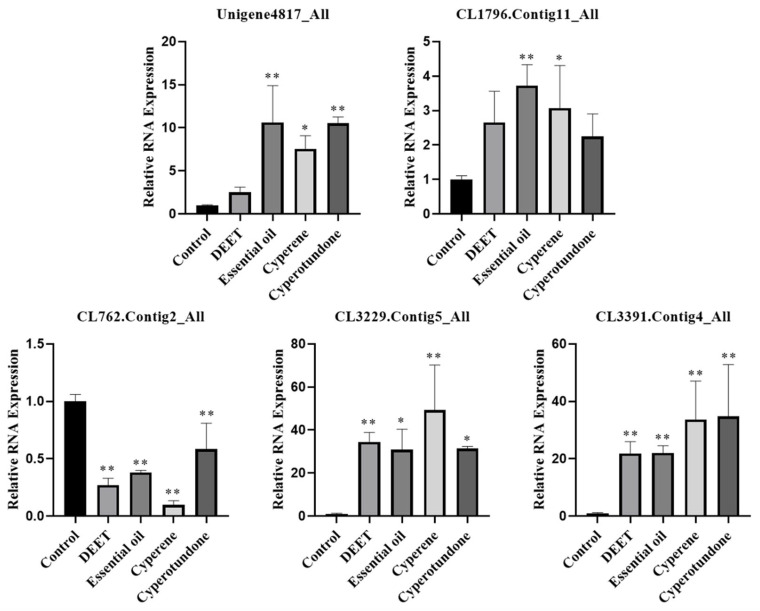
qRT-PCR analysis results (mean ± standard deviation) of the relative RNA expression of odorant receptor genes in *T. confusum* treated with different substances. *, significant difference in the relative RNA expression of odorant receptor genes in pests between the treatment groups and the control groups (* *p* < 0.05; ** *p* < 0.01).

In the present research, quantitative real-time polymerase chain reaction (qRT-PCR) analyses were conducted. The results demonstrated significant changes in the expression levels of the five odorant receptor genes in *T. confusum* when exposed to the EO, its primary compounds cyperene and cyperotundone, and the positive control, DEET. As depicted in Figure 6, all tested samples significantly upregulated the expression of the genes CL3229.Contig5_All and CL3391.Contig4_All (*p* < 0.05). The EO and at least one of its main compounds also significant upregulated (*p* < 0.05) the expression of Unigene4817_All and CL1796.Contig11_All. Conversely, the same treatment significantly downregulated the expression of the gene CL762.Contig2_All (*p* < 0.05).

These findings provide insights into the molecular mechanisms by which the EO and its constituents may influence olfactory reception in the pest, potentially offering new avenues for pest management strategies.

## 3. Discussion

GC-MS analysis revealed that the EO of *C. esculentus* roots, obtained by steam distillation, contained sesquiterpenoids cyperene and cyperotundone as the main compounds. The pest repellent efficacy of the EO and its two main compounds were more enduring than that of the positive control, DEET (Table 2, Figure 3). DEET is a well-established insect repellent. Nonetheless, some studies have reported negligible impacts of DEET on pest mortality rates [20]. Despite this, DEET has exhibited a significantly high repellency effect and has been employed as a positive control in repellent efficacy tests on *Tribolium* species [21].

Cyperotundone demonstrated longer-lasting activity compared cyperene, aligning with its reduced volatility and extended RI in GC-MS analysis. Cyperotundone also exhibited enhanced bioactivity against *T. confusum*, suggesting that oxygenated compounds may possess superior repellency. The main compounds, cyperotundone and cyperene, were as effective as or more potent than the EO itself in pest repellency. Consequently, the bioactivity of *C. esculentus* root EO might be mainly attributed to these two compounds.

In the context of evaluating the efficacy of repellents against *Tribolium* species, it is imperative to consider the collective behavior of these insects rather than isolating individual specimens, as *Tribolium* species exhibit aggregative behavior, which significantly influences their response to repellent stimuli [22]. The current study utilized a group-based assay to assess the repellent efficacy, thereby providing a more accurate simulation of real-world pest behavior.

AChE, the enzyme responsible for the hydrolysis of acetylcholine, and GST, a key detoxification enzyme, plays key roles during pest physiological and detoxification process [15,23]. Inhibition of AChE in pests causes the accumulation of acetylcholine and results in paralysis or death of the pests. On the other hand, GST facilitates the detoxification of toxins by conjugating with electrophilic molecules and hydrolyzing ester bonds, thereby neutralizing the harmful effects of various poisons. The inhibition of GST, therefore, impairs the pest’s detoxification system. The inhibitory effects of the EO and its compounds on these enzymes suggest their potential to harm the pest.

Odorant receptors (ORs), integral to the peripheral olfactory system, are signaling proteins that play a pivotal role in detecting and responding to chemical cues from the environment. These receptors are particularly significant in the context of pest control, as they can be targeted by odor molecules present in EOs, which may interact with them to elicit a repellent effect on pests [5,24]. Pests’ ORs typically have a structural domain with seven α-helical transmembrane domains, a C-terminal located outside the cell, and an N-terminal in the cytoplasm [25,26,27]. The binding of odor molecules to ORs activates a signaling cascade involving Gαolf, Adcy3, and cAMP, which opens CNG ion channels, playing a crucial role in the initial stages of odor detection [24]. Due to the lack of specific recognition of odorants in most insects, an odorant receptor can generally be activated by multiple ligands, and the same ligand can also activate multiple receptors [5]. These characteristics are consistent with the current research findings that each of the two major constituent compounds, cyperene and cyperotundone, from the EO of *C. esculentus* roots could interfere with several odorant receptors. Furthermore, both compounds were capable of interfering with some of the same odorant receptors, highlighting the complexity and potential of these natural compounds in pest control strategies. In recent years, researchers have identified specific OR genes in *T. confusum* that play a significant role in responding to limonene. The knockdown of the TconOR93 gene resulted in a decreased repellent rate, indicating that this gene is a major effector in the perception of limonene [5]. This finding suggests that distinct OR genes can discriminate between responses to specific compounds. The specific OR genes that serve as major effectors in the perception of cyperene and cyperotundone, which are the predominant compounds in the EO studied in this manuscript, warrant further investigation.

Cyperene and cyperotundone were recently reported to have insecticidal effects on *Aphis craccivora* and *Planococcus lilacinus*, and cyperotundone was found to be inhibitory on GST and AChE in the pests [15]. This study discovered the repellent activity of cyperene and cyperotundone against *T. confusum*, and for the first time revealed that these two components could not only inhibit the activity of GST and AChE, but also interfere with the expression of odorant receptor genes in the pest, adding new information for our understanding of the pesticide/repellent mechanisms of these natural sesquiterpenoids. Olfactory receptor genes might be involved in the perception of the EO and its main compounds by pests, thereby mediating the spatial repellent effects of the EO and its compounds on pests.

Besides cyperene and cyperotundone, other chemical constituents in the EO might contribute to the pest repellent activity. For instance, cubebol and caryophyllene oxide have been reported to contribute to bioactivities of other EOs. A study by Basile et al. [28] evaluated the insecticidal activity of EOs from *Calendula incana* and *Laserpitium siler* against stored product pests, identifying cubebol as a major constituent in *C. maritima* EO, which exhibited higher biocidal activity. This aligns with our findings, suggesting that cubebol, despite being a minor compound, could play a role in the observed pest repellent activity. Similarly, caryophyllene oxide, another minor compound in our study, has been reported to exhibit synergistic repellent and irritant effects when mixed with vetiver oil against mosquito vectors [29]. This underscores the potential of caryophyllene oxide to enhance the efficacy of our EO mixture against a broader range of insect species.

While our study focuses on the bioactivity of EO compounds, it is important to consider the broader ecological implications. Basile et al. [28] discussed the potential of *C. maritima* oil as a promising candidate for further tests as an alternative biocide, emphasizing the need for green systems in pest control. This aligns with our study’s aim to explore natural alternatives that are more environmentally friendly. The potential for resistance development in insect populations to these compounds is a valid concern, especially given the widespread use of synthetic chemicals. As Nararak et al. [29] suggested, understanding the repellent activity of compounds like caryophyllene oxide was crucial in developing new strategies for mosquito control. Our study contributes to this knowledge by identifying the potential of more compounds in EOs to contribute to pest control strategies.

## 4. Materials and Methods

### 4.1. Plant, Pest, Reagents, and Instruments

The whole plants of *C. esculentus* were collected from Ordos (40°45′ N 109°29′ E), Inner Mongolia, China in Oct 2021 and botanically identified by the authors. A voucher specimen (NPFFCEL-2022-5) was deposited in School of Life Sciences, Inner Mongolia University. The plant was dried and divided to leaves, stems, roots and tubes (Figure S1). The roots were rinsed with clean water and dried before being pulverized and extracted.

The pests naturally occurred during the storage of flaxseed kernel in a cabinet of 25 ± 3 °C and were identified as *Tribolium Confusum* through morphological observation and DNA sequence comparison (Figures S8 and S9 and Tables S6 and S7). The pests were moved to plastic boxes containing flour and reared in dark at room temperature of 25 ± 3 °C, and humidity of 65 ± 5% before use.

Pure water was from Guangzhou Watsons Food & Beverage Co., Ltd. (Guangdong, China). Analytical-grade ethanol was from XiLong Chemical Co., Ltd. (Guangdong, China). Analytical-grade N, N-Diethyl-3-methylbenzamide (DEET) was from Shanghai Pesticide Research Institute Co., Ltd. (Shanghai, China). Analytical-grade coomassie brilliant blue G-250 and reduced glutathione were from Sigma–Aldrich Co., (Shanghai, China). Analytical-grade 5,5′-Dithio bis-(2-nitrobenzoic acid) (DTNB) and acetylthiocholine were from Macklin Co., Ltd. (Shanghai, China). Analytical-grade 1-Chloro-2,4-dinitrobenzene (CDNB) was from Aladdin Biochemical Technology Co., Ltd. (Shanghai, China).

Nuclear magnetic resonance (NMR) spectra were measured using an ASCEND 600 instrument from Brucker, Switzerland. GC–MS analyses were carried out using an Agilent GCMS-7890B-5977B Ultra system (Agilent, Santa Clara, CA, USA).

### 4.2. Extraction of the Essential Oil and Analyses of the Constituent Compounds

*C. esculentus* roots (5 kg) were divided into five portions and extracted with steam distillation at 95 ± 5 °C for 5 h each to yield 0.55 ± 0.018 g of an EO at a yield of 0.055 ± 0.0018%.

The volatile compounds in the EO were analyzed using an Agilent GC-MS with electron impact ionization for the detection. A DB-wax-fused silica capillary column (30 m × 0.25 mm i.d., 0.25 μm film thickness) with a stationary phase of 5% phenyl-polymethylsiloxane at a film thickness of 0.25 μm was used for chromatographic separation. The injector, ion source and quadrupole temperatures were set at 260 °C, 230 °C and 150 °C, respectively, the mass scan range was set between *m*/*z* 20 and 400, and the electric potential was set to 70 eV. Helium was used as the carrier gas, with a velocity of 1 mL/min. The injection was performed at a temperature of 260 °C. The temperature program of the GC oven was set as follows: it initially had a temperature of 40 °C for 5 min, was heated at a rate of 5 °C/min to 220 °C, followed by a heating rate of 20 °C/min to 250 °C, and then held at this temperature for 5 min. The obtained mass spectra and RIs with respect to a homologous series of n-alkanes from Sigma-Aldrich were compared with those in the MS databases (NIST Chemistry WebBook, SRD 69) to identify the constituent compounds. The relative contents were calculated by peak area normalization using the formula X_ij_′ = 100·X_ij_/(∑^n^_k−1_·X_kj_), where X_ij_′ is the normalized peak area of the *i*th peak in the *j*th profile, X_ij_ is the peak area of the *i*th peak in the *j*th profile, and n is the total number of peaks in the profile. The sum-normalized data were multiplied by 100 and are expressed in terms of their percent contribution to the total area. The analyses were performed for three times, and the results are expressed as mean ± standard deviation.

### 4.3. Isolation of the Major Chemical Constituents from the EO of C. esculentus Roots

The steam-distillated extract (2.75 g) of *C. esculentus* roots was subjected to column chromatography on silica gel (200–300 mesh), using a glass column with a diameter of 3 cm and a height of 35 cm, and eluted with a hexane-ethyl acetate solvent system. The hexane-ethyl acetate 99:1 eluate was subjected to sublimation to obtain compound **8**. The hexane-ethyl acetate 50:50 eluate (approximately 38 mg after solvent evaporation) was further separated using preparative silica gel thin layer chromatography on a SIL GF 254 plate (Qingdao Marine Chemical Co., Ltd., Qingdao, China) with a plate format of 20 × 20 cm and a layer thickness of 1 mm. The mobile phase, consisting of hexane-ethyl acetate in a 20:1 ratio, was contained within a glass chamber measuring 22.5 × 22.5 × 7 cm for the development of the plate. After development for 1 h, the plate was examined under UV light at 254 nm. The dark band was scraped off and transferred to a funnel containing filter paper, and then washed with hexane-ethyl acetate (50:50 ratio) to obtain compound **41** after solvent evaporation.

### 4.4. Assessment of Pest Repellent Activity

Both acetone and ethanol have been reported as solvents used in testing for pest-repellent activity [30,31]. In this experiment, the EO of *C. esculentus* roots and the isolated compounds were dissolved in ethanol at 5.000, 2.500, 1.250, 0.625 and 0.3125 mg/mL, and the repellent activities were assessed based on the method reported by Caballero-Gallardo et al. [32] with modifications. Circular Whatman No. 1 filter paper with a diameter of 8.6 cm was cut in half from the middle. Amounts of 500 μL of the sample solutions were applied to one half of the filter papers, resulting in concentrations of 86.12, 43.06, 21.53, 10.76 and 5.38 μg/cm^2^ (μg per square centimeter filter paper). An equal volume of ethanol (as a control) was applied to the other half of each filter paper. In the positive control group, 99.0% DEET was applied in place of the sample solution. After the filter paper was air-dried for 1 min to allow the solvent to evaporate, the two halves were reattached using adhesive tape and then placed in 9 cm glass Petri dishes. Adult *T. confusum* beetles, at approximately one month post-emergence from the pupal stage and from a mixed population without sex selection, were used for the experiment. Twenty adult *T. confusum* were put to the center of each dish, and the dishes were covered and placed in darkness at 24–26 °C. The numbers of *T. confusum* present on the extract and control halves of the filter paper were recorded after 0.5 to 24 h of exposure. Percentage repellency for a given exposure interval was calculated as percentage repellency = [(Nc − Ns)/(Nc + Ns)] × 100, where Nc and Ns are the number of pests on the control and sample areas, respectively. Five replicates were performed for each concentration of the samples.

### 4.5. Assessment of the Inhibitory Effects on the Pest Enzymes, Acetylcholinesterase (AChE) and Glutathione S-transferase (GST)

The enzyme-inhibitory activities against AChE and GST in *T. confusum* were evaluated using the method reported by Singh et al., 2024 [15], with some modifications. The experiment was performed on 9 cm glass Petri dishes containing filter paper, with one half being applied with test samples and the other half with ethanol as a control. After the solvent evaporated, the pests were exposed to various concentrations of the samples for 24 h in the same manner as described in Section 4.4. Subsequently, the insects from each group were combined with 0.1 M phosphate buffer (pH 7.4) at a ratio of 1:100 (insect weight in grams to buffer volume in milliliters). The mixtures were homogenized using a high-speed tissue grinder at temperatures below 4 °C, followed by centrifugation at 4 °C at 9500× *g* for 30 min. The supernatant was then subjected to 0.22 μm microfiltration, and the resulting filtrates were used to determine protein concentration and enzyme activities.

Protein concentrations were determined using the Bradford assay [33] by combining 20 μL of pest homogenate with 200 μL of Coomassie Brilliant Blue G-250. The resulting mixtures were incubated at 37 °C for 15 min, after which the absorbance at 595 nm was measured. The assay was performed in triplicate, and the mean values were employed for the estimation of protein concentrations

For the AChE activity assay, 100 μL of the pest homogenate was mixed with 50 μL of DTNB-acetylthiocholine 1:1. The reaction mixture was incubated at 37 °C for 30 min, and the absorbance at 410 nm was measured. The molar absorption coefficient of 13.6 × 10^3^ L/mol/cm for 5-mercapto-nitrobenzoic acid (TNB) [34] was used for calculating AChE activity and the results were expressed as milliunits per milligram of protein (mU/mg).

For the GST activity assay, 10 μL of the pest homogenate was mixed with 90 μL of phosphate buffer (0.1 M, pH 7.4)-reduced glutathione-CDNB 98:1:1. The mixture was incubated at 37 °C for 20 min, and the absorbance at 340 nm was measured. The molar absorption coefficient of 9.6 × 10^3^ L/mol/cm for CDNB [35] was used for calculating GST activity and the result was expressed as mU/mg.

### 4.6. Molecular Docking Experiment

Based on the analysis by Liao et al. [5], the amino acid sequences of five olfactory receptor genes (Unigene4817_All, CL1796.Contig11_All, CL762.Contig2_All, CL3229.Contig5_All, CL3391.Contig4_All) in *Tribolium confusum* were determined. Protein structures were predicted using AlphaFold2 reported by Jumper et al. [36], with homologous sequence alignments generated through MMseqs2 [37,38]. Due to their similarity to previously reported proteins from T. castaneum, these receptors were designated as TconOR93, TconOR139, TconOR94a, TconOR1, and TconOR22c-like [5]. The structures of compounds **8**, compounds **41**, and DEET were obtained from PubChem database (https://pubchem.ncbi.nlm.nih.gov, accessed on 13 September 2024). Protein and ligand preparation was performed using AutoDockTools 1.5.7, followed by molecular docking using AutoDock Vina 1.1.2 [39]. The docking results were then analyzed for chemical bonds using LigPlot+ (version 2.2.4), and visualized using PyMOL 3.0 [40].

### 4.7. qRT-PCR Experiments

The expression of 5 genes related to the odorant receptors [5] of *T. confusum* were analyzed by qRT-PCR. In the sample groups, the pests were treated with 86.12 μg/cm^2^ of DEET, EO, cyperene or cyperotandone for 24 h. Untreated pests were used as control. Total RNA was extracted from the pests using TRIzol (TransGen Biotech, Beijing, China), and RNA concentrations were determined using NanoDrop One (Thermo Fisher Scientific, Madision, WI, USA). The literature-reported [5] primers (Table S8) were synthesized by Sangon Biotech Co., Ltd. (Shanghai, China), and used for the qRT-PCR analysis of these genes. Glyceraldehyde-3-phosphate dehydrogenase (GAPDH) gene was used as an internal reference. Referring to the manual of TransScript One-Step gDNA Removal and cDNA Synthesis SuperMix (TransGen Biotech, Beijing, China), 1 μg total RNA and a 20 μL reverse transcription system were used to synthesize cDNA with SimpliAmp^TM^ Thermal Cycler (Thermo Fisher Scientific, Madision, WI, USA). The cDNA was diluted threefold and used as a template to perform qRT-PCR using LightCycler 480 II (F. Hoffmann-La Roche Ltd., Basel, Switzerland). The reaction mixture contained 10 μL PerfectStart Green qPCR SuperMix (TransGen Biotech, Beijing, China), 0.8 μL forward and 0.8 μL reverse gene-specific primer, 2 μL cDNA template, and 6.4 μL nuclease-free water. The mixture was heated to 95 °C for 2 min, followed by 40 cycles of 95 °C for 10 s and 60 °C for 20 s. For each independent biological duplicate, three technical replicates were performed. The relative expression levels were analyzed using 2^−ΔΔCT^.

### 4.8. Statistical Analysis

The pest-repellent activities were assessed in five replicates for each concentration of each sample, and the results are presented as mean ± standard deviation. To compare the effects of different samples at the same concentration and exposure interval, the results of 5 repeated experiments were analyzed by one-way ANOVA followed by Tukey’s honestly significant difference (HSD) test using GraphPad Prism 8.0.2, with percentage repellency as an evaluation variable and samples as factors.

The enzyme activities and gene expressions were assessed in triplicated experiments, with data analyzed by one-way ANOVA followed by Tukey’s HSD test using GraphPad Prism 8.0.2. For the pest enzyme assay, the evaluation variables were enzyme activities and the factors were sample concentrations or sample treatment groups. For the gene expression, the evaluation variables were relative gene expressions and the factors were sample treatment groups. Differences were considered significant at *p* < 0.05, and *p* < 0.01.

## 5. Conclusions

In conclusion, the essential oil of *C. esculentus* root exhibited strong and long-lasting repellent activities against the grain pest *T. confusum*. Sesquiterpenoids such as cyperene and cyperotundone are the primary compounds responsible for the pest-repellent activity of the essential oil. The essential oil, cyperene, and cyperotundone all demonstrated inhibitory effects on the activity of glutathione S-transferase and acetylcholinesterase in the pest. Odorant receptors may play a role in the response of *T. confusum* to the essential oil and the two compounds. The essential oil extracted from *C. esculentus* roots, along with its principal compounds, demonstrated superior pest repellent efficacy compared to DEET, underscoring their potential as natural alternatives in integrated pest management strategies.

## Figures and Tables

**Table 1:** Components and their relative contents in the essential oil of *C. esculentus* roots.

Number	Constituents	Structural Type	RIcalc ^a^	RIdb ^b^	RA ^c^
**1**	*α*-Pinene	Monoterpenoid	1023	1016	0.18 ± 0.03
**2**	*β*-Pinene	Monoterpenoid	1099	1096	0.56 ± 0.06
**3**	Limonene	Monoterpenoid	1188	1189	0.06 ± 0.01
**4**	1,8-Cineole	Monoterpenoid	1199	1199	0.14 ± 0.02
**5**	*p*-Cymene	Monoterpenoid	1258	1269	0.10 ± 0.01
**6**	Cyprotene	Sesquiterpene analog	1428	-	0.42 ± 0.06
**7**	*α*-Copaene	Sesquiterpenoid	1485	1488	0.98 ± 0.14
**8**	Cyperene	Sesquiterpenoid	1522	1527	13.54 ± 0.17
**9**	Cypera-2,4-diene	Sesquiterpenoid	1533	-	0.65 ± 0.11
**10**	Pinocarvone	Monoterpenoid	1552	-	0.61 ± 0.03
**11**	Myrtenal	Monoterpenoid	1611	1626	0.61 ± 0.01
**12**	Rotundene	Sesquiterpenoid	1630	1629	5.30 ± 0.05
**38**	Pinocarveol	Monoterpenoid	1642	1651	1.18 ± 0.03
**14**	*trans*-Verbenol	Monoterpenoid	1667	1673	0.86 ± 0.04
**15**	*γ*-Muurolene	Sesquiterpenoid	1678	1681	0.20 ± 0.02
**16**	Verbenone	Monoterpenoid	1690	1701	0.27 ± 0.02
**17**	*δ*-Guaiene	Sesquiterpenoid	1707	1642	0.72 ± 0.07
**18**	Muurolene	Sesquiterpenoid	1714	1720	0.10 ± 0.04
**19**	Carvone	Monoterpenoid	1718	1740	0.35 ± 0.04
**20**	Myrtenol	Monoterpenoid	1779	1792	1.12 ± 0.07
**21**	Ar-himachalen-2-ol	Sesquiterpenoid	1788	-	1.09 ± 0.14
**22**	Calamenene	Sesquiterpenoid	1820	1832	0.37 ± 0.13
**23**	Cyperene epoxide	Sesquiterpenoid	1854	-	3.99 ± 0.14
**24**	*α*-Calacorene	Sesquiterpenoid	1900	1916	0.28 ± 0.08
**25**	Aristolene epoxide	Sesquiterpenoid	1920	-	1.76 ± 0.02
**26**	Cubebol	Sesquiterpenoid	1931	1914	0.33 ± 0.01
**27**	Caryophyllene oxide	Sesquiterpenoid	1971	1974	3.35 ± 0.18
**28**	Longifolenaldehyde	Sesquiterpenoid	2005	-	3.38 ± 0.04
**29**	Spathulenol	Sesquiterpenoid	2025	2088	1.77 ± 0.03
**30**	3,4,8,8-Tetramethyl-4,5,6,7,8,8a- hexahydro-1H-3a,7-methanoazulen-4-ol	Sesquiterpenoid	2049	-	0.89 ± 0.06
**31**	Aristolone	Sesquiterpenoid	2087	2284	4.84 ± 0.08
**32**	15-Hydroxy-3-copaene	Sesquiterpenoid	2121	-	3.06 ± 0.45
**33**	Lemnalol	Sesquiterpenoid	2136	2135	0.91 ± 0.06
**34**	6-Isopropenyl-4,8a-dimethyl-1,2,3,5,6,7,8,8a-octahydro-naphthalen-2-ol	Sesquiterpenoid	2145	-	2.86 ± 0.04
**35**	Cyperadione	Sesquiterpenoid	2165	-	2.18 ± 0.11
**36**	2H-Cycloprop [c]indene-2,3 (3ah)-dione, hexahydro-3a,7,7-trimethyl-	Sesquiterpene analog	2190	-	1.64 ± 0.19
**37**	Cadalene	Sesquiterpenoid	2202	2200	0.38 ± 0.07
**38**	2,5-Di-tert-butyl-1,4-benzoquinone	Aromatic	2207	-	3.54 ± 0.54
**39**	Globulol	Sesquiterpenoid	2225	-	1.94 ± 0.15
**40**	Longiverbenone	Sesquiterpenoid	2245	2254	2.96 ± 0.14
**41**	Cyperotundone	Sesquiterpenoid	2263	-	13.50 ± 0.64
**42**	*α*-Cyperone	Sesquiterpenoid	2271	-	1.21 ± 0.37
**43**	*γ*-Gurjunenepoxide-(2)	Sesquiterpenoid	2282	-	2.80 ± 0.31
**44**	Zizanal	Sesquiterpenoid	2289	-	1.47 ± 0.09
**45**	8-Oxo-9H-cycloisolongifolene	Sesquiterpenoid	2308	-	2.54 ± 0.04
**46**	7R,8R-8-Hydroxy-4-isopropylidene-7-methylbicyclo [5.3.1]undec-1-ene	Sesquiterpenoid	2345	-	1.96 ± 0.42
**47**	*β*-Betulenol	Sesquiterpenoid	2357	-	1.88 ± 0.36
**48**	Costol	Sesquiterpenoid	2420	2606	1.16 ± 0.03
**49**	Aristolochene	Sesquiterpenoid	2470	-	0.84 ± 0.03
**P1** ^d^	Phthalic Acid Diisobutyl Ester	Aromatic	2522	2526	0.84 ± 0.28
**P2** ^d^	Dibutyl phthalate	Aromatic	2677	2680	1.30 ± 0.08
**50**	Palmitic acid	Fatty acid	2896	2899	1.00 ± 0.05

The chemical components were first tentatively identified by comparing the mass spectra with the GC-MS compound library. Retention indices (RIs) were determined relative to a homologous series of n-alkanes. ^a^ RIcalc, calculated RI; ^b^ RIdb, RI from database (NIST Chemistry WebBook, SRD 69). For compounds with reported RIdb values, their identities were confirmed by comparing the RIcalc with RIdb. Compounds marked with “-” in the RIdb column were identified only by the GC-MS compound library, as no reported RI data were available for these compounds using a DB-wax column. The presence of the main compounds, cyperotundone and cyperene, was confirmed by NMR. ^c^ RA, mean relative area of chromatographic peak (%) ± SD (n = 3); ^d^ plasticizer that may come from centrifuge tubes or other containers, rather than plant-based ingredients.

**Table 2:** Percentage repellency against *T. confusum* of *C. esculentus* root essential oil and the main components.

	Concentration (μg/cm^2^)	Exposure Interval (h)						
		0.5	1	2	4	8	16	24
Essential oil	86.12	60.0 ± 7.1 ^a^	70.0 ± 12.3 ^abc^	84.0 ± 11.4 ^a^	80.0 ± 10.0 ^a^	76.0 ± 8.9 ^a^	68.0 ± 8.4 ^a^	62.0 ± 11.0 ^a^
Cyperene	86.12	48.0 ± 19.2 ^a^	56.0 ± 5.5 ^ac^	74.0 ± 8.9 ^a^	76.0 ± 11.4 ^a^	69.0 ± 8.9 ^a^	58.0 ± 13.0 ^ab^	48.0 ± 13.0 ^a^
Cyperotundone	86.12	52.0 ± 8.4 ^a^	60.0 ± 7.1 ^ac^	70.0 ± 14.1 ^a^	82.0 ± 14.8 ^a^	74.0 ± 11.4 ^a^	68.0 ± 16.4 ^a^	64.0 ± 15.2 ^a^
DEET	86.12	82.0 ± 8.4 ^b^	88.0 ± 13.0 ^ab^	90.0 ± 10.0 ^a^	78.0 ± 11.0 ^a^	52.0 ± 27.8 ^a^	38.0 ± 23.9 ^b^	32.0 ± 40.9 ^a^
Essential oil	43.06	56.0 ± 13.4 ^a^	64.0 ± 15.2 ^a^	74.0 ± 5.8 ^a^	78.0 ± 13.0 ^a^	66.0 ± 8.9 ^a^	54.0 ± 11.4 ^ab^	44.0 ± 15.2 ^a^
Cyperene	43.06	44.0 ± 8.9 ^a^	52.0 ± 11.0 ^a^	68.0 ± 13.0 ^a^	64.0 ± 16.7 ^a^	56.0 ± 15.2 ^a^	52.0 ± 8.4 ^ab^	44.0 ± 16.7 ^a^
Cyperotundone	43.06	48.0 ± 21.7 ^a^	56.0 ± 11.4 ^a^	62.0 ± 11.0 ^a^	80.0 ± 15.8 ^a^	60.0 ± 12.3 ^a^	64.0 ± 15.2 ^a^	54.0 ± 15.2 ^a^
DEET	43.06	60.0 ± 7.1 ^a^	68.0 ± 8.4 ^a^	72.0 ± 8.4 ^a^	64.0 ± 11.4 ^a^	32.0 ± 44.4 ^a^	26.0 ± 30.5 ^b^	22.0 ± 59.3 ^a^
Essential oil	21.53	50.0 ± 18.7 ^a^	56.0 ± 16.7 ^a^	62.0 ± 13.0 ^a^	66.0 ± 11.4 ^ab^	56.0 ± 11.4 ^ab^	40.0 ± 31.3 ^ab^	26.0 ± 30.5 ^a^
Cyperene	21.53	36.0 ± 5.48 ^a^	48.0 ± 13.0 ^a^	44.0 ± 16.7 ^a^	64.0 ± 11.4 ^ab^	56.0 ± 13.4 ^ab^	46.0 ± 8.9 ^ab^	42.0 ± 13.0 ^a^
Cyperotundone	21.53	42.0 ± 11.0 ^a^	58.0 ± 11.0 ^a^	60.0 ± 18.7 ^a^	70.0 ± 12.3 ^a^	76.0 ± 16.7 ^a^	66.0 ± 15.2 ^a^	48.0 ± 16.4 ^a^
DEET	21.53	24.0 ± 46.7 ^a^	36.0 ± 35.1 ^a^	44.0 ± 16.7 ^a^	34.0 ± 32.1 ^b^	26.0 ± 25.1 ^b^	4.0 ± 56.8 ^b^	2.0 ± 48.7 ^a^
Essential oil	10.76	24.0 ± 16.7 ^a^	30.0 ± 15.8 ^a^	38.0 ± 30.3 ^a^	42.0 ± 19.2 ^a^	32.0 ± 31.1 ^a^	26.0 ± 30.5 ^ab^	2.0 ± 51.7 ^a^
Cyperene	10.76	30.0 ± 14.1 ^a^	36.0 ± 11.4 ^a^	56.0 ± 13.4 ^a^	46.0 ± 15.2 ^a^	44.0 ± 16.7 ^a^	36.0 ± 15.1 ^ab^	30.0 ± 18.7 ^a^
Cyperotundone	10.76	32.0 ± 16.4 ^a^	46.0 ± 15.2 ^a^	48.0 ± 13.0 ^a^	60.0 ± 14.1 ^a^	68.0 ± 8.4 ^a^	56.0 ± 11.4 ^a^	44.0 ± 16.7 ^a^
DEET	10.76	22.0 ± 23.9 ^a^	30.0 ± 25.5 ^a^	26.0 ± 37.8 ^a^	38.0 ± 44.9 ^a^	−4.0 ± 78.3 ^a^	−2.0 ± 49.7 ^b^	−2.0 ± 73.3 ^a^
Essential oil	5.38	18.0 ± 34.9 ^a^	30.0 ± 48.5 ^a^	34.0 ± 28.8 ^a^	24.0 ± 51.8 ^a^	36.0 ± 30.5 ^a^	22.0 ± 43.2 ^a^	6.0 ± 56.8 ^a^
Cyperene	5.38	10.0 ± 57.0 ^a^	14.0 ± 8.9 ^a^	28.0 ± 21.7 ^a^	28.0 ± 21.7 ^a^	26.0 ± 19.5 ^a^	12.0 ± 63.4 ^a^	4.0 ± 47.2 ^a^
Cyperotundone	5.38	20.0 ± 20.0 ^a^	30.0 ± 35.4 ^a^	38.0 ± 21.7 ^a^	44.0 ± 20.7 ^a^	38.0 ± 25.9 ^a^	28.0 ± 27.8 ^a^	20.0 ± 37.4 ^a^
DEET	5.38	22.0 ± 55.4 ^a^	30.0 ± 55.2 ^a^	20.0 ± 74.8 ^a^	50.0 ± 52.0 ^a^	10.0 ± 48.5 ^a^	22.0 ± 47.6 ^a^	−8.0 ± 57.6 ^a^

Values are mean ± standard deviation; DEET (N, N-diethyl-3-methylbenzamide), positive control. ^a, b, c^, different letters indicate significant differences (*p* < 0.05) by one-way ANOVA followed by Tukey’s HSD test between sample groups at the same concentration and exposure interval. In the absence of significant insecticidal activity, pests tend to approach each other. For example, in one test, all pests stayed in the sample treatment area, while in another test, all pests stayed in the control area, resulting in a large standard deviation.

**Table 3:** Docking of EO main compounds and DEET with odorant receptor proteins in the pest.

Protein	Compound	Binding Energy (kcal/mol)	Hydrogen Bonding Residue (Bond length)
TconOR93	Cyperene	−7.9	
TconOR93	Cyperotundone	−7.9	LYS-146 (2.87 Å)
TconOR93	DEET	−7.0	
TconOR139	Cyperene	−7.5	
TconOR139	Cyperotundone	−8.2	
TconOR139	DEET	−6.8	
TconOR94a	Cyperene	−6.9	
TconOR94a	Cyperotundone	−7.2	
TconOR94a	DEET	−6.0	
TconOR1	Cyperene	−7.7	
TconOR1	Cyperotundone	−7.2	ASN-188 (2.92 Å)
TconOR1	DEET	−5.7	THR (2.81 Å)
TconOR22c-like	Cyperene	−6.7	
TconOR22c-like	Cyperotundone	−6.6	
TconOR22c-like	DEET	−5.6	

## Data Availability

The data presented in this study are available on request from the corresponding author. Supplementary Materials is available at Molecules online.

## Supplementary Materials

The following supporting information can be downloaded at: https://www.mdpi.com/article/10.3390/molecules30030631/s1, Figure S1: The whole plant and different parts of *Cyperus esculentus*; Figure S2: Representative photographs illustrating the pest-repellent effects of different parts of *C. esculentus* after an 8-h exposure; Figure S3: ^1^HNMR spectrum of cyperene; Figure S4: ^1^H-^1^H COSY spectrum of cyperene; Figure S5: ^1^HNMR spectrum of cyperotundone; Figure S6: ^1^H-^1^H COSY spectrum of cyperotundone; Figure S7: Analysis of variance results; Figure S8: PCR process diagram; Figure S9: Gel electrophoresis of the PCR products of *Tribolium confusum* DNA; Table S1: Percentage preference of *T. confusum* to different parts of *C. esculentus*; Table S2: Comparison of the ^1^HNMR data of compounds **8** and **41** with literature reported data, Table S3: Three-way ANOVA of concentration, time and substance for the repellent activity; Table S4: Inhibitory activities on the pest enzymes AChE and GST; Table S5: qRT-PCR analysis results of the relative RNA expression of odorant receptor genes in *T. confusum* of different treatment groups; Table S6: PCR amplification system; Table S7: NCBI database alignment results; Table S8: Primers for RT-qPCR analysis of odorant receptor gene levels expressed in *T. confusum*.
