# Supplementary material for: Main Chemical Components, Activity and Mechanism of Repellence of Cyperus esculentus Essential Oil Against Tribolium confusum
Source: Molecules. 2025 Jan 31;30(3):631. doi: 10.3390/molecules30030631 (PMC11820371; doi:10.3390/molecules30030631)
Supplement: Supplementary file 1 [file molecules-30-00631-s001.zip › molecules-3347783-supplementary.pdf]

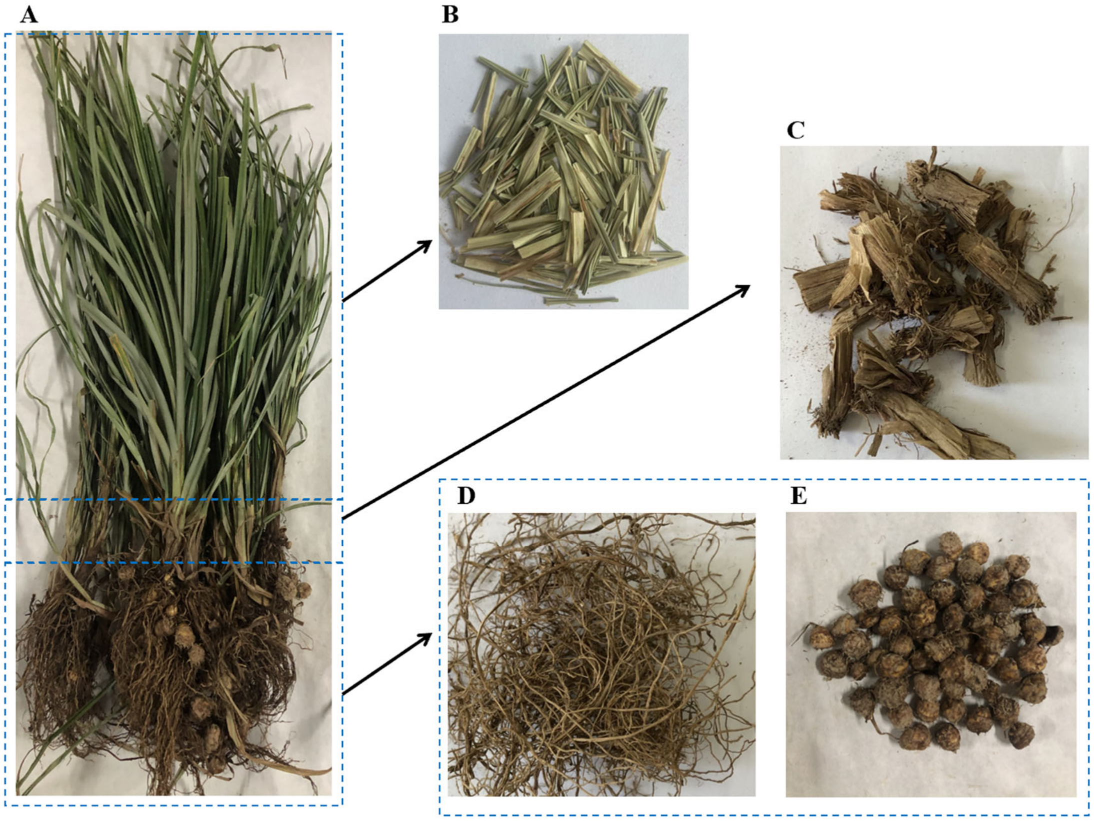

Figure S1. The whole plant and different parts of *Cyperus esculentus*. (**A**) The whole plant; (**B**) The leaves; (**C**) The stems; (**D**) The roots; (**E**) The tubers.

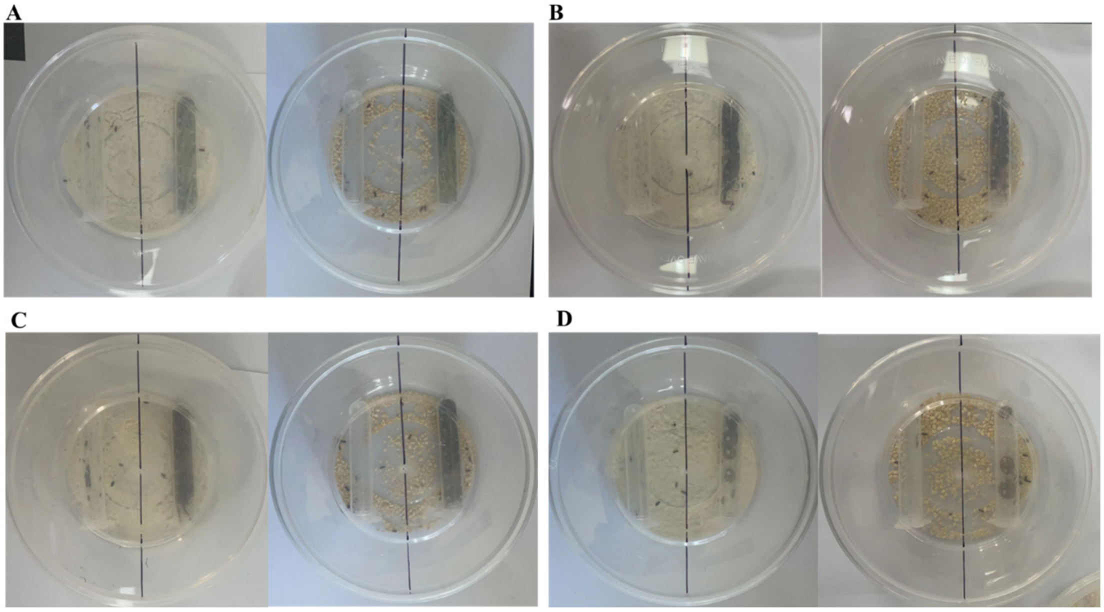

Figure S2 Representative photographs illustrating the pest-repellent effects of different parts of *C. esculentus* after an 8-hour exposure. Some pests are observed hiding under the centrifuge tubes. (**A**) The leaves in flour (left) and flaxseed kernel meal (right); (**B**) The stems in flour (left) and flaxseed kernel meal (right); (**C**) The roots in flour (left) and flaxseed kernel meal (right); (**D**) The tubers in flour (left) and flaxseed kernel meal (right).

Flour or flaxseed kernels (1.00 g ± 0.03 g) were evenly spread on the bottom of a disposable lunch box (diameter 14 cm, height 4 cm). *C. esculentus* were separated into different parts: leaves, stems, roots, and tubers (Figure S1). Each part was weighed to 1.00 g ± 0.03 g and placed into 10 mL centrifuge tubes that were evenly punctured with 55 holes using a steel needle. In each lunch box containing flour or flaxseed kernel meal, two test tubes were placed as far away as possible: one containing *C. esculentus* and the other serving as an empty control (Figure S2). Twenty *T. confusum* were placed in the middle of each lunch box. After the lunch box was covered, the number of pests on both sides was recorded at different times. The repellency activity was calculated using the following formula: Percentage repellency = [(Nc - Ns) / (Nc + Ns)] × 100%. Where, Nc is the number of pests on the control side, and Ns is the number of pests on the sample side. These experiments were conducted in five independent replicates.

Table S1. Percentage preference of *T. confusum* to different parts of *C. esculentus*

	Exposure interval (h)					
	1	2	4	8	16	24
Leaves in Flour	-22.0±21.75^{aA}	12.0±21.7^{aA}	2.0±19.2^{aA}	-6.0±16.7^{bcA}	4.0±23.0^{abA}	8.0±21.7^{aA}
Leaves in Flax[a]	14.0±8.9^{aA}	-2.0±40.2^{aA}	2.0±25.9^{aA}	-2.0±34.2^{bcA}	2.0±25.9^{abA}	4.0±32.9^{aA}
Stems in Flour	-12.0±17.9^{aA}	18.0±21.7^{aA}	-12.0±22.8^{aA}	-14±23.0^{bcA}	-12.0±16.4^{abA}	-30.0±21.2^{aA}
Stems in Flax	2.0±22.8^{aA}	4.0±35.1^{aA}	-14.0±36.5^{aA}	-18.0±40.9^{bcA}	8.0±22.8^{abA}	10.0±21.2^{aA}
Roots in Flour	-14.0±23.0^{aD}	-8.0±13.0^{aCD}	18.0±8.4^{aBC}	48.0±8.4^{aA}	32.0±8.4^{aAB}	26.0±15.2^{aAB}
Roots in Flax	-10.0±7.1^{aB}	8.0±8.4^{aBC}	22.0±13.0^{aAC}	32.0±13.0^{aAC}	34.0±18.2^{aA}	24.0±11.4^{aAC}
Tubers in Flour	6.0±18.2^{aA}	-4.0±35.1^{aA}	2.0±19.2^{aA}	10.0±18.7^{acA}	-20.0±45.8^{bA}	0.0±61.2^{aA}
Tubers in Flax	18.0±31.1^{aA}	0.0±29.2^{aA}	8.0±25.9^{aA}	2.0±17.9^{acA}	10.0±15.8^{abA}	10.0±23.5^{aA}

[a]Flaxseed kernel meal.

Values are mean ± SD of five replicates. Different letters indicate significant differences ($p<0.05$) by one-way ANOVA followed by Tukey's HSD test. a, b, c, d, comparison between different sample groups of the same exposure interval; A, B, C, D, comparison between different exposure interval of the same sample groups.

Table S2. Comparison of the ^{1}H-NMR data of compounds **8** and **41** with literature reported data

position	**cyperene (8)**		**cyperotundone (41)**	
	^{1}HNMR measured	^{1}HNMR from literature [1]	^{1}HNMR measured	^{1}HNMR from literature [2]
2	1.68 m; 1.44 overlapped	1.68; 1.44	1.97 d, 16.5; 2.10 d, 16.5	2.00 d, 17.5; 2.14 d, 17.5
3	2.63 m; 2.20 overlapped	2.69; 2.21		
6	2.20 overlapped; 1.79 br d, 17.0	2.17; 1.78	2.55 dd, 19.1, 6.6; 2.26 d, 19.1	2.60 dd, 19.0, 6.5; 2.29 d, 19.0
7	1.86 overlapped	1.81	1.92 m	1.96 ddd, 6.5, 3.5, 2.5
8	1.87 m; 1.30 m	1.83; 1.27	1.92 m; 1.37 m	1.95 ddd 13.0, 13.0, 2.5; 1.41 ddd, 13.0, 6.5, 3.5
9	1.43 overlapped; 1.12 m	1.39; 1.12	1.57 m; 1.00 m	1.59 dt, 15.0, 6.5; 1.01 dddd, 15.0, 13.0, 13.0, 6.5
10	1.97 m	1.93	2.14 m	2.17 d penta, 13.0, 6.5
12	0.97 s	0.97	1.07 s	1.10 s
13	0.79 s	0.81	0.71 s	0.74 s
14	1.64 s	1.62	1.68 s	1.72 s
15	0.83 d, 6.8	0.86	0.58 d, 6.6	0.67 d, 6.5

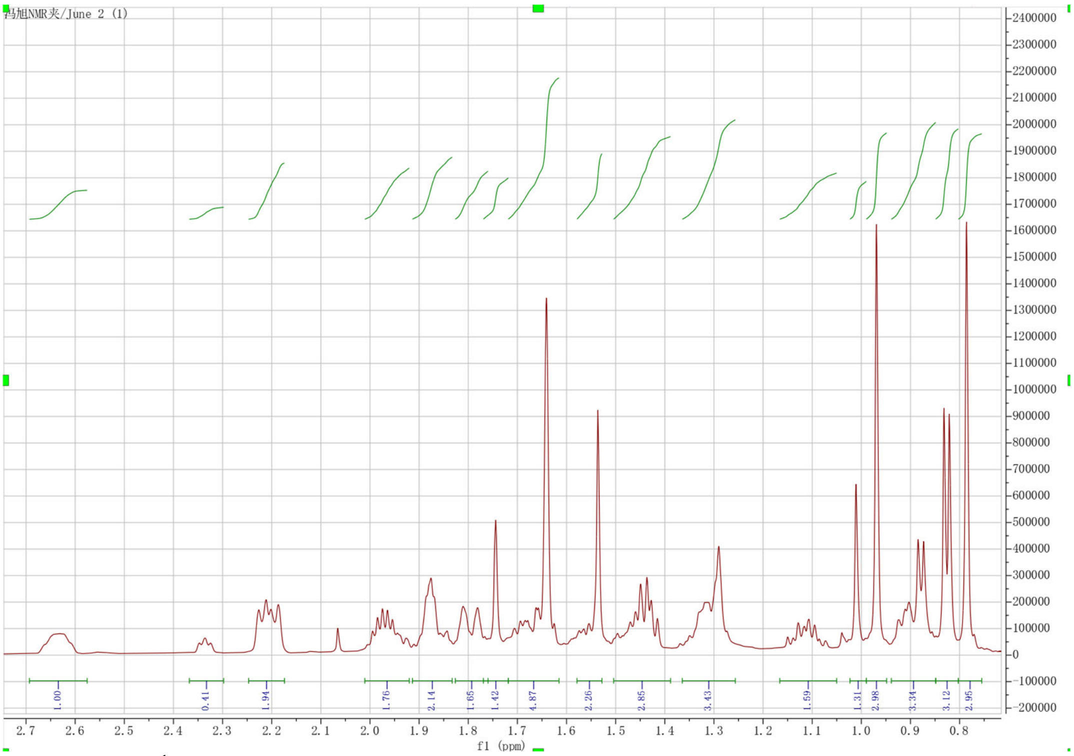

Figure S3. ^{1}HNMR spectrum of cyperene

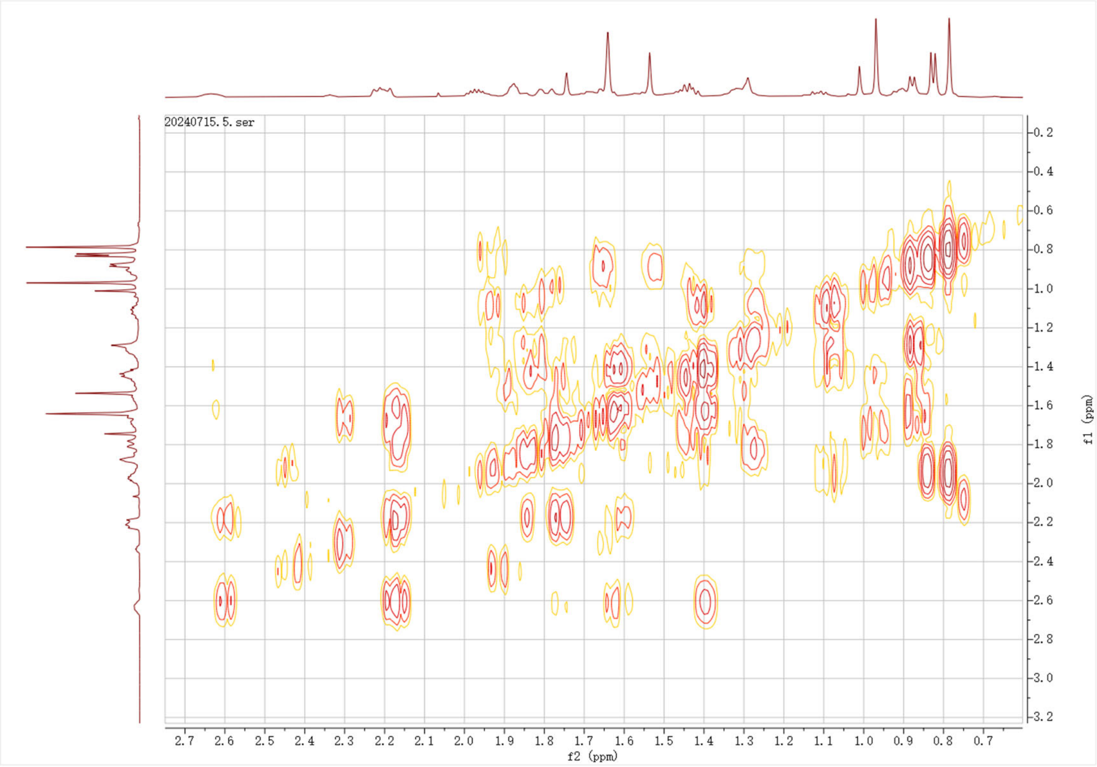

Figure S4. ^{1}H-^{1}H COSY spectrum of cyperene

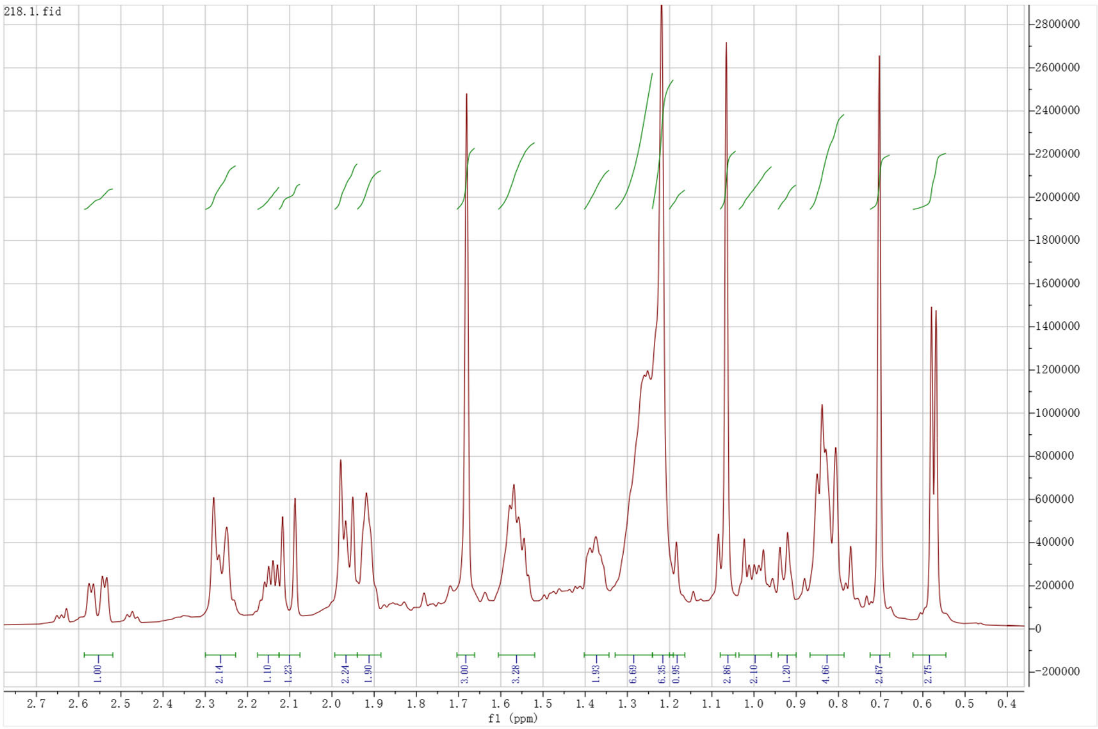

Figure S5. ^{1}HNMR spectrum of cyperotundone

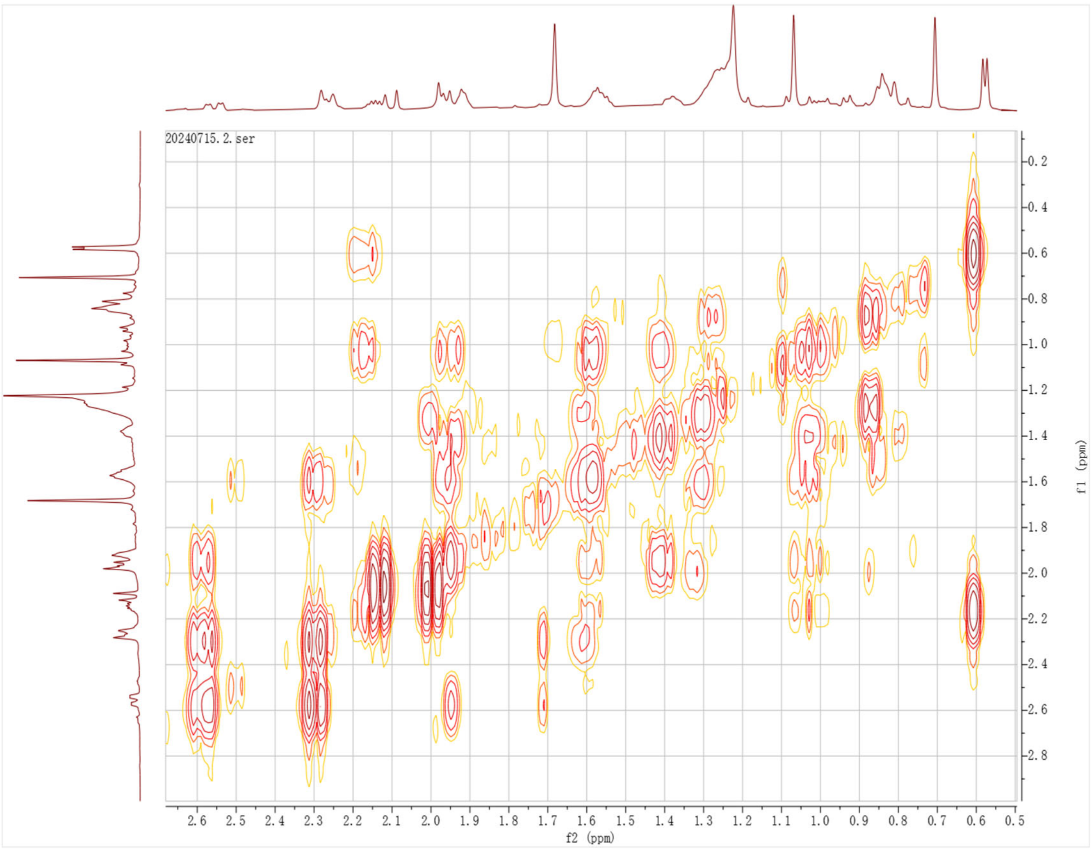

Figure S6. ^{1}H-^{1}H COSY spectrum of cyperotundone.

In the study investigating the effects of time, substance and concentration concentration on pest repellent activity (expressed as repellency %), a three-way analysis of variance (ANOVA) was employed. The results, as presented in Table S3, indicated that all three factors—concentration ($F=51.459$, $p=0.000 < 0.05$), time ($F=12.399$, $p=0.000 < 0.05$), and substance ($F=13.365$, $p=0.000 < 0.05$)—exhibited statistical significance, suggesting the presence of main effects and that each factor differentially influences the repellent activity.

The interaction between time and substance was found to be significant ($F=2.290$, $p=0.002 < 0.05$), indicating that the effect of time on repellent activity is moderated by the substance. Similarly, the interaction between concentration and substance was also significant ($F=2.576$, $p=0.002 < 0.05$), which implies that the influence of concentration on repellent activity varies across different substances.

Upon examining the mean comparison plots (Fig S7), it was observed that the repellent activity of *C. esculentus* EO and its main compounds was superior to that of DEET in terms of low concentration and temporal efficacy. This suggests that *C. esculentus* EO and the main compounds may offer more effective protection against the pests over time and at varying concentrations compared to the synthetic repellent DEET.

Table S3. Three-way ANOVA of concentration, time and substance for the repellent activity.

Difference source	SS	DF	MS	F	p	(Partial η^2)
Intercept	1428772.321	1	1428772.321	1773.142	0.000**	0.760
Concentration	165858.571	4	41464.643	51.459	0.000**	0.269
Time	59943.929	6	9990.655	12.399	0.000**	0.117
substance	32307.821	3	10769.274	13.365	0.000**	0.067
Concentration &Time	2921.429	24	121.726	0.151	1.000	0.006
Concentration & Substance	24907.714	12	2075.643	2.576	0.002**	0.052
Time & Substance	33220.929	18	1845.607	2.290	0.002*	0.069
Concentration & Time & Substance	14752.286	72	204.893	0.254	1.000**	0.032
Residual	451240.000	560	805.786			

R^2: 0.425. * $p<0.05$, ** $p<0.01$.

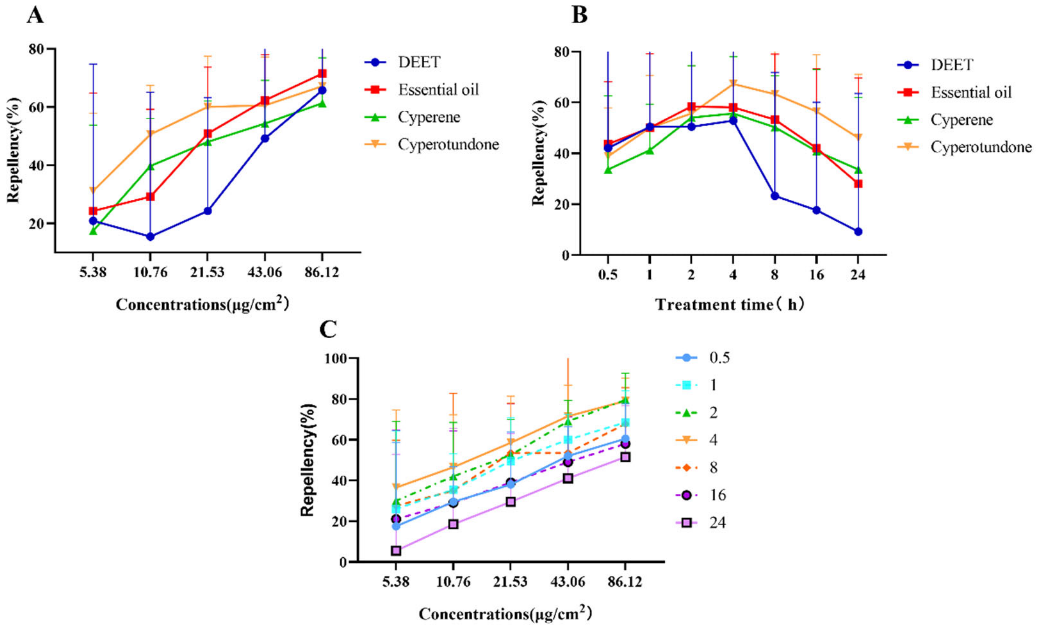

Figure S7. Analysis of variance results. (**A**) Comparison of concentration and group for repellency; (**B**) Comparison of time and group for repellency; (**C**) Comparison of time and concentration for repellency.

Table S4. Inhibitory activities on the pest enzymes AChE and GST

Concentration (μg/cm^2)	AChE（mU/mg）				GST（mU/mg）			
	DEET	Essential oil	Cyperene	Cyperotundone	DEET	Essential oil	Cyperene	Cyperotundone
86.12	1.36±0.04^{aA}	1.20±0.03cA^{aB}	1.29±0.01^{aA}	1.18±0.01^{aB}	17.13±1.34^{aB}	15.68±0.93cA^{aB}	24.30±0.86^{aA}	22.45±0.74^{aA}
43.06	1.38±0.02^{aA}	1.28±0.01bA^{bC}	1.33±0.01^{bB}	1.24±0.01^{aD}	21.67±0.80^{bB}	17.30±0.93bA^{abC}	27.83±1.40^{bA}	23.71±0.47^{aB}
21.53	1.40±0.03^{aA}	1.29±0.01bb^{cB}	1.37±0.02^{bA}	1.30±0.02^{abB}	27.42±0.90^{cC}	19.45±0.89bb^{bD}	31.78±0.26^{cA}	23.75±0.34^{aB}
10.67	1.51±0.03^{bA}	1.36±0.03b^{cB}	1.49±0.02^{cA}	1.32±0.02^{bB}	32.85±0.98^{dA}	22.25±0.92b^{cC}	34.30±1.05^{cdA}	26.79±0.77^{bB}
0	1.60±0.02^{c}	1.60±0.02^{d}	1.60±0.02^{d}	1.60±0.02^{c}	36.67±1.20^{e}	36.67±1.20^{d}	36.67±1.20^{d}	36.67±1.20^{c}

Values are mean ± standard deviation. Different letters indicated the existence of significantly difference by one-way ANOVA followed by Tukey's HSD test ($p < 0.05$). a, b, c, d, comparison between different concentrations of the same sample; A, B, C, D, comparison between different samples of the same concentration.

Table S5. qRT-PCR analysis results of the relative RNA expression of odorant receptor genes in *T. confusum* of different treatment groups.

Gene	Control	DEET	Essential oil	Cyperene	Cyperotundone
Unigene4817_All	1.00±0.06	2.50±0.62	10.61±4.28**	7.54±1.53*	10.52±0.74**
CL1796.Contig11_All	1.00±0.11	2.66±0.90	3.73±0.60**	3.08±1.23*	2.25±0.66
CL762.Contig2_All	1.00±0.06	0.27±0.06**	0.38±0.02**	0.10±0.04**	0.58±0.23**
CL3229.Contig5_All	1.03±0.30	34.49±4.42**	30.91±9.49*	49.35±20.85**	31.35±1.08*
CL3391.Contig4_All	1.02±0.21	21.68±1.36**	22.05±2.51**	33.46±12.79**	34.84±17.97**

Values are mean ± standard deviation. *, significant difference in the relative RNA expression of odorant receptor genes in pests between the treatment groups and the control groups (* $P < 0.05$; ** $P < 0.01$).

Identification of *Tribolium Confusum* through DNA sequence comparison

Reagents: Analytical grade Tris-saturated phenol and isoamyl alcohol were from Macklin Co., Ltd (Shanghai, China). Analytical grade 1-Bromo-3-chloropropane was from Seven Biotech Co., Ltd (Beijing, China). Proteinase K for DNA extraction and reagents for PCR reaction system were form TransGen Biotech Co. Ltd (Beijing, China). The primers were synthesized by Sango Biotech Co., Ltd (Shanghai, China).

"DNA Extraction: Five of the pests were transferred to a sterile Eppendorf (EP) tube and homogenized using a high-throughput tissue homogenizer at 990 r/s for 10 seconds. Subsequently, 190 μL of tissue lysis buffer and 10 μL of proteinase K solution were added. The mixture was incubated overnight in a 56 °C water bath and then centrifuged at 12,000 g for 5 minutes. The supernatant was transferred to a new tube, and an equal volume of DNA extraction solution (tris-saturated phenol: 1-bromo-3-chloropropane: isoamyl alcohol in a ratio of 25:24:1) was added. The mixture was vortexed for 15 seconds, left to stand at room temperature for 5 minutes, and then centrifuged at 12,000 g for 10 minutes. The supernatant was transferred to a new tube and mixed with an equal volume of isopropanol, then incubated at 4 °C for 10 minutes before centrifuging at 12,000 g for 10 minutes to sediment the white DNA pellets. The supernatant was carefully removed, and the DNA pellet was washed with 1 mL of 75% ethanol, centrifuged at 12,000 g for 10 minutes, and the ethanol was discarded. The EP tube was inverted on absorbent paper to air-dry and to avoid cross-contamination. The DNA was dissolved in 200 μL of ddH_2O and stored at -20 °C until analysis."

PCR amplification: PCR amplification was carried out using High-Taq enzyme, with the specific conditions detailed in Figure S7 and Table S3. The forward and reverse primers were BF1-CO1: ACWGGWTGRACWGTNTAYCC; BR2-CO1: TCDGGRTGNCCRAARAAYCA.

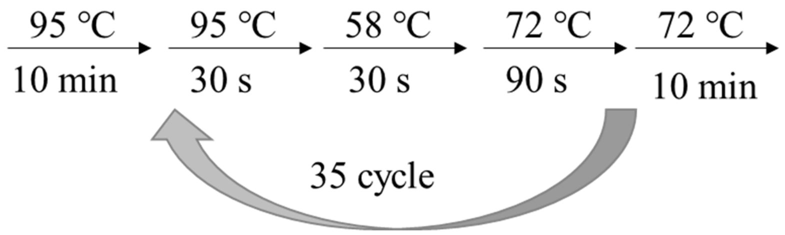

Figure S8. PCR process diagram

Table S6. PCR amplification system

Components	Volume（μL）
Buffer	10
dNTP	8
DNA	2
BF1-CO1	1
BF2-CO1	1
High-Fi Taq	1
dd H_2O	37

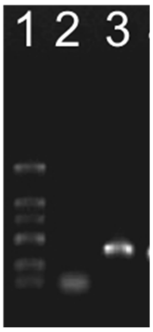

Figure S9. Gel electrophoresis of the PCR products of *Tribolium confusum* DNA. Lane 1, Marker (from top to bottom: 2000 bp, 1000 bp, 750 bp, 500 bp, 250 bp, 100 bp). Lane 2, negative control (dd H_2O). Lane 3, the sequence after amplification.

Table S7. NCBI database alignment results.

No.	Description	Scientific Name	Max Score	Toal Score	Query Cover	Per. Ident	Accession
1	*Tribolium confusum* voucher O12 cytochrome oxidase subunit 1(CO1) gene.	*Tribolium confusum*	523	523	100 %	100 %	MK120453.1
2	*Tribolium confusum* voucher FDA:CFSAN CO21-002 cytochrome oxidase subunit I (COl) gene.	*Tribolium confusum*	523	523	100 %	100 %	MG458957.1
3	*Tribolium confusum* mitochondrion,complete genome.	*Tribolium confusum*	523	523	100 %	100 %	NC_026702.1
4	*Tribolium confusum* voucher ZMUO<FIN>:006460 cytochrome oxidase	*Tribolium confusum*	523	523	100 %	100 %	KJ964296.1

	subunit 1 (COl) gene.						
5	*Tribolium confusum* voucher FHI-015 cytochrome oxidase subunit 1 (COl) gene.	*Tribolium confusum*	523	523	100 %	100 %	HM398858.1

The sequencing results were processed using Mega 11 software to remove low-quality areas and outlier sites. The data were upload to NCBI database for comparison and the top 5 Query Cover results are shown in the Table S5. The results confirmed that the pest was *Tribolium confusum*.

Table S8. Primers for RT-qPCR analysis of odorant receptor gene levels expressed in *T. confusum*.

Gene ID	Sequences (Forward)	Sequences (Reverse)
Glyceraldehyde-3-phosphate dehydrogenase (*GAPDH*)	GGCGATGTTAAAGCTGAAGG	AGGCTTTTTCAATGGTGGTG
Unigene4817_All	AGGTGCTGTATGTGGATGT	GTAAACAATGCTCCCAACCA
CL1796.Contig11_All	TTTCTCCTGATGTGTGGG	CCTTGAGCTTCTCCAAAC
CL762.Contig2_All	GAGAGTGTCCATCGGTATT	GCAGGTGTAGTTGAAATGAG
CL3229.Contig5_All	CAATGTGGTCGCTTGAAC	GGTTGATACTGAACGGATAC
CL3391.Contig4_All	CCTCTTGTGCTGCGATT	GAAACCACCCATCAGGAATA

References

1. Joseph-Nathan, P.; Martinez, E.; Santillan, R.L.; Wesener, J.R.; Gunther, H. Two-Dimensional NMR Studies of Cyperene. *Org. Magn. Reson*. **1984**, *22*, 308-311. http://doi.org/10.1002/mrc.1270220507.
2. Xu, Y.; Zhang, H.-W.; Wan, X.-C.; Zou, Z.-M. Complete assignments of ^{1}H and ^{13}CNMR data for two new sesquiterpenes from *Cyperus rotundus* L. *Magn. Reson. Chem*. **2009**, *47*, 527–531. http://doi.org/10.1002/mrc.2416.
